# Tumor Resection Guided by Intraoperative Indocyanine Green Dye Fluorescence Angiography Results in Negative Surgical Margins and Decreased Local Recurrence in an Orthotopic Mouse Model of Osteosarcoma

**DOI:** 10.1245/s10434-018-07114-9

**Published:** 2018-12-27

**Authors:** Adel Mahjoub, Alejandro Morales-Restrepo, Mitchell S. Fourman, Jonathan B. Mandell, Lu Feiqi, Margaret L. Hankins, Rebecca J. Watters, Kurt R. Weiss

**Affiliations:** 1 School of Medicine, University of Pittsburgh, Pittsburgh, PA USA; 2 Musculoskeletal Oncology Laboratory, Department of Orthopaedic Surgery, University of Pittsburgh, Pittsburgh, PA USA; 3 School of Medicine, Tsinghua University, Beijing, China; 4 Department of Pharmacology and Chemical Biology, University of Pittsburgh, Pittsburgh, PA USA

## Abstract

**Background:**

Surgical resection with negative margins is the foundation of extremity sarcoma management. Failure to achieve negative surgical margins can result in local recurrence (LR), a potentially devastating complication. Indocyanine green (ICG) is a US FDA-approved fluorophore previously used to guide carcinoma resections. We investigated the potential of ICG as an intraoperative guide during experimental sarcoma resection.

**Methods:**

Fifty 6-week-old immunocompetent Balb/c female mice received left proximal tibia paraphyseal injections of 5 × 10^5^ K7M2 murine osteosarcoma cells. Animals were separated into two groups (*n* = 25 each): (1) ICG-assisted surgical resection; and (2) no ICG-assisted resection. Resections were performed 4 weeks after primary tumor engraftment. All animals received 7.5 ug ICG via retro-orbital injection 12 h prior to surgery. ICG fluorescence measurements and clinical evaluations were performed 4 weeks after resection to detect LR.

**Results:**

Eleven of 25 animals from each group developed gross tumors. Four weeks after resection, group 1 had 0/11 tumor recurrences, while group 2 had recurrences in 9/11 (81.8%) experimental mice (*p* < 0.0002) (Fig. 2). There was a 100% NPV in group 1, and no tumor recurrence with fluorescence-free margins after the primary surgery. Group 2 had a 100% positive predictive value for the development of an LR if any fluorescent signal was present at the surgical margin after resection.

**Conclusion:**

Intraoperative ICG guidance led to reliably negative surgical margins and a diminished LR rate. Given the benign safety profile of ICG and its prior clinical success, these results could be immediately translatable to the clinical realm.

Extremity sarcomas are rare and aggressive mesenchymal tumors that account for approximately 1% of adult malignancies and 7% of pediatric malignancies. Surgery with negative histological margins remains the foundation of sarcoma management. Incomplete surgical resections and positive margins can result in local recurrence (LR) rates as high as 40%.^1^–^8^ LR is a potentially devastating complication that can necessitate additional surgery and treatment, added morbidity, increased cost, and potential loss of limb.^2^^,^^3^,^8^–^11^ In osteosarcoma (OS), the most common primary malignancy of bone, LR portends a very poor prognosis.^1^,^3^,^12^–^14^

The intraoperative determination of a surgical margin’s adequacy is based on the individual surgeon’s judgment and experience, which has been shown to be unreliable and subjective. Intraoperative histopathology is also subjective, time-consuming, and unable to assess the adequacy of sarcoma margins intraoperatively in real time. Intraoperative technologies capable of ensuring the complete surgical removal of sarcomas could potentially decrease LR and would thus be of tremendous comprehensive benefit.

Indocyanine green (ICG) dye is a US FDA-approved, non-toxic, near-infrared fluorophore. After intravenous injection it binds tightly to plasma albumin, thereby confining it to the intravascular space. After injection, ICG is rapidly metabolized by the liver and only remains in areas of disorganized vasculature, such as in burned or traumatized tissue or within tumors. After the initial washout phase, the dye only persists in these areas and it thus yields an essentially binary measurement, adding to its utility. The propensity of ICG to extravasate from areas of disorganized or ‘leaky’ vasculature, such as burns or within tumors, has been previously utilized to identify and resect hepatocellular carcinomas.^15^–^17^ Additional work by our group has noted the detection of ICG signal in primary and metastatic experimental OS tumors. In addition to visual detection, we observed the precise histological localization of ICG to experimental tumors.^15^ These findings suggest that ICG fluorescence angiography may illuminate tumor margins when utilized intraoperatively, thus helping sarcoma surgeons to avoid positive margins.^15^

We therefore hypothesized that ICG fluorescence angiography can be used as an accurate, real-time, intraoperative margin detector during the surgical removal of sarcoma in a previously validated mouse model of OS. We further hypothesized that surgical resections that utilize ICG guidance would have a lower LR rate than resections performed based on surgeon judgment alone.

## Methods

**Fig. 1:**
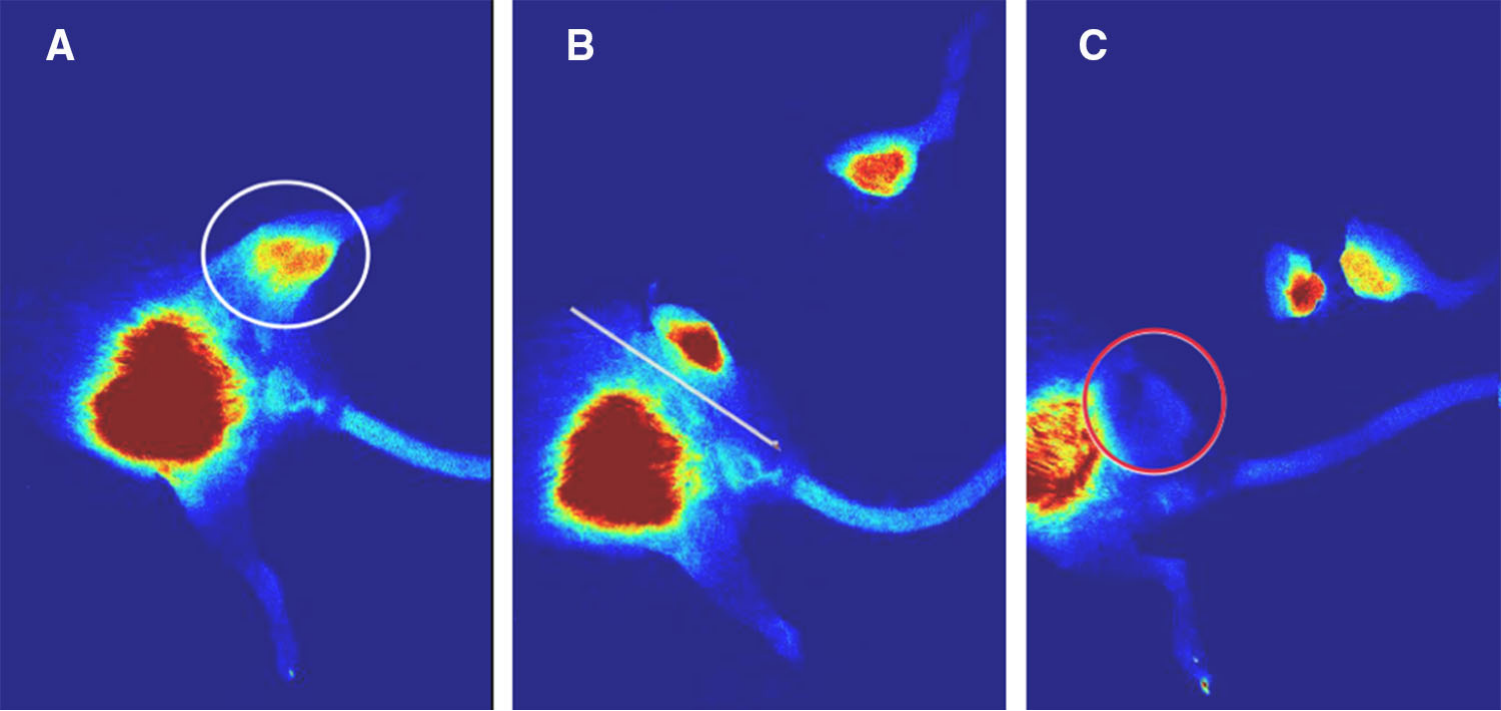
Stepwise removal of positive tumor margins under indocyanine-green guidance. **a** Preoperative fluorescent image is seen using near infra-red. Fluorescent signal is seen in the left proximal hind limb (circled). **b** Positive fluorescent signal remains in the left proximal hind limb after initial resection. **c** No area of fluorescence left behind in the left proximal hind limb. A clear surgical margin can be seen, with no fluorescence in the resected left proximal hind limb (circled)

After approval by the University of Pittsburgh Institutional Animal Care and Use Committee (IACUC Protocol# 16119548), fifty 6-week-old immunocompetent Balb/c female mice received left proximal tibia paraphyseal injections of 5 × 10^5^ K7M2 murine OS cells, as has been previously described.^15^,^18^ Mice were then prospectively separated into two groups (*n* = 25 each): (1) ICG-assisted surgical resection; and (2) no ICG-assisted resection. After tumor engraftment, transfemoral amputations were performed 4 weeks after the initial injection to simulate surgical resection with negative margins. Twelve hours prior to surgery, all animals received 7.5 ug of ICG via retro-orbital injection. All ICG measurements were performed using the SPY-Elite System (Stryker Corporation, Kalamazoo, MI, USA) as previously described. The timing and dosage of ICG administration was also based on our previous work.^15^,^18^ Surgical resections in group 1 were performed under the direct guidance of ICG–infrared imaging until there was no longer any fluorescent signal in the remaining hind limb (Fig. 1). Group 2 surgical resections were performed without ICG-fluorescence assistance. Notation was made of group 2 animals with positive ICG signals after amputation. Animals were clinically monitored for LR, and ICG fluorescent measurements were performed 4 weeks after surgery to detect LR. Gross tumor recurrence, positive predictive value (PPV), and negative predictive value (NPV) were observed and calculated.

## Results

**Fig. 2:**
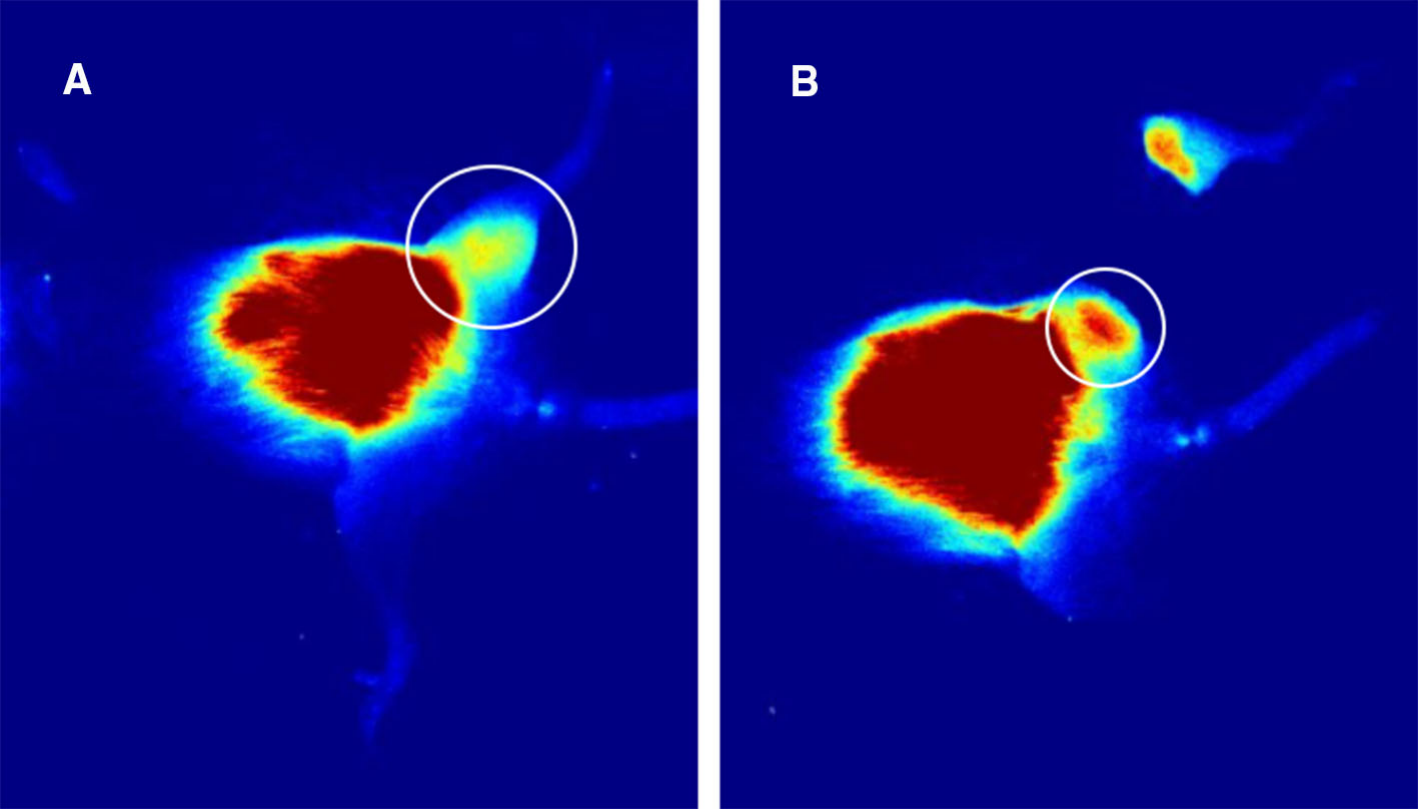
Residual tumor remains in the non-ICG-guided tumor resection at the end of surgery. **a** Preoperative fluorescent image shows a positive fluorescent signal in the left hind limb. **b** Non-ICG-guided resection shows a positive fluorescent signal in the residual left hind limb at the end of surgery. *ICG* indocyanine-green

At the time of tumor resection (4 weeks after OS cell injection), 11/25 animals from each group had palpable tumor growth. Intraoperatively, ICG signal was clearly detected within the experimental sarcomas (Fig. 1), as has been our experience. In both experimental groups, ICG signal was clearly present or absent at the surgical margin at the conclusion of amputation surgery (Figs. 1, 2). ICG signal was a clearly discernable intraoperative guide for group I (ICG guidance) animals. In group 2, residual ICG signal after amputation was noted in 9 of 11 animals.

**Fig. 3:**
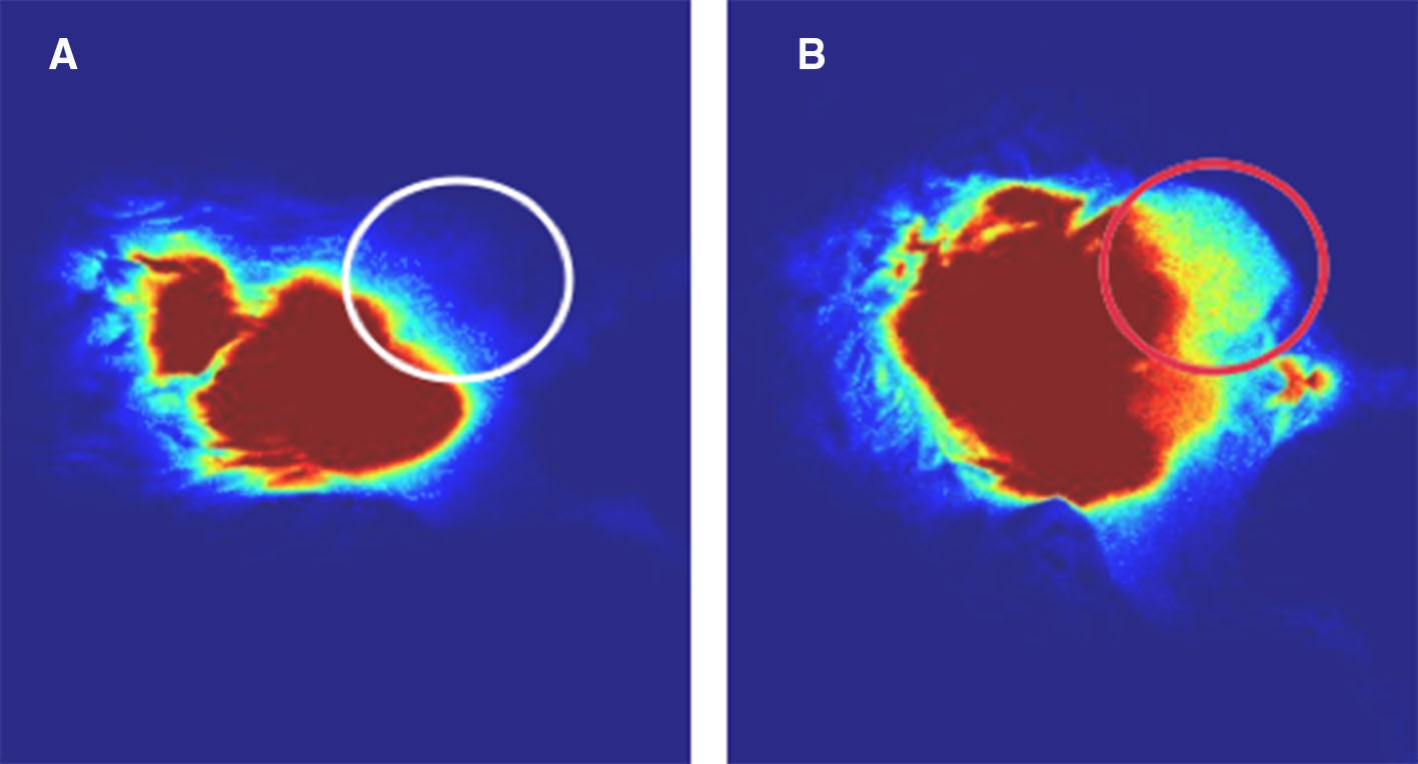
Local recurrence assessed 4 weeks postoperatively after primary sarcoma resection of the left proximal hind limb with and without ICG guidance. **a** ICG-guided resection with no local recurrence and lack of fluorescent signal at the residual left proximal hind limb (circled). **b** Non-ICG-guided resection shows local recurrence with positive fluorescent signal at the residual left proximal hind limb (circled red). *ICG* indocyanine-green

Four weeks after surgery (8 weeks after OS cell injection), animals were evaluated both clinically and with SPY imaging. Group 1 had 0/11 LRs, whereas group 2 had LR in 9/11 animals, detectable both clinically and with ICG testing (81.8%; *p* < 0.0002) (Fig. 3). The use of ICG in group 1 had a 100% NPV, such that tumors did not recur if all fluorescent signal was removed during the primary surgery. Group 2 demonstrated a 100% PPV for the development of an LR if any fluorescent signal was present at the surgical margin after resection. The two animals in group 2 without fluorescent signal after tumor removal also had a 100% NPV for not developing an LR.

## Discussion

Margin-negative surgery is foundational in the care of extremity sarcoma patients. The assessment of any given surgical margin’s adequacy is subjective, complex, and multifactorial. It is affected by the amount and type of tissue through which the surgical plane passes, the efficacy of any neoadjuvant treatment (external radiation therapy and/or chemotherapy), the experience level of the operative surgeon, and other factors.^2^,^4^,^5^,^9^,^11^,^19^–^25^ Variables not related to the surgery itself include tissue desiccation and retraction of the specimen once it is removed, sampling error that results in false negative results, and the experience level of the histopathologist.

LR is a catastrophic complication that can necessitate additional surgery, including limb loss, additional treatments, and possible mortality.^8^,^10^,^19^,^26^ In a previously described preclinical model of OS, we demonstrated that ICG near-infrared fluorescence angiography signal localized precisely to primary and metastatic OS tumors and not the adjacent microenvironment.^15^ These observations suggested that ICG might present a viable intraoperative adjunct for obtaining negative surgical margins, thus decreasing the rate of LR.

In this study, we describe the use of ICG as an intraoperative guide for the removal of OS tumors in our validated orthotopic mouse model. We found that ICG indeed provided a useful intraoperative guide for the removal of OS tumors. The observation of residual intraoperative ICG signal yielded 100% positive NPVs for LR. These findings suggest that ICG may indeed be of benefit in sarcoma surgery, particularly for histological subtypes such as myxofibrosarcomas that have notoriously high LR rates.^6^,^27^

This study has many limitations. The experimental design lends itself to inherent experimental bias. The authors may have performed a more thorough surgical resection in group 1 animals (ICG-assisted) and may have been unintentionally less careful with group 2 animals that did not have ICG guidance. Although this is a valid concern, we raise two points that may alleviate the impact of this potential bias. First, the intent of the experiment was to determine if ICG can successfully illuminate areas of residual sarcoma in vivo and thus guide the surgeon intraoperatively. As Fig. 1 dramatically illustrates, we found this to be the case. Second, the work described here is meant to serve as a proof of concept that stimulates further investigation of this technology in sarcoma. As surgeons do not possess the ability to see and feel very small tumor volumes, there is certainly equipoise to suggest that additional studies are warranted.

Second, the present study only utilized one histological subtype of sarcoma (OS), a disease entity comprised of over 50 distinct histologies.^24^,^27^,^28^ This was done mainly out of expediency as our model of OS and the use of ICG to detect primary and metastatic tumors have already been well-characterized.^15^,^18^ Additional testing in other histologies besides OS would strengthen our hypothesis.

Third, this study was performed in mice. Although ours is an immunocompetent model, it is not guaranteed that human sarcomas will behave in exactly the same way as in our mouse model.

We did not employ intraoperative frozen section in these experiments as it was not experimentally feasible. Even so, intraoperative frozen section is not ubiquitously utilized in orthopedic oncology due to variability in resources and access to dedicated musculoskeletal pathologists at different institutions. This is a potential strength of the described technology—that ICG may be a real-time, objective tool to assist the surgeon that does not rely on an additional source of potential human error. The purpose of this study was to see if ICG might assist the surgeon’s eyes and hands intraoperatively. Although beyond the scope of this work, a comparison of ICG with frozen section is a potential area for future research.

Although our previous work^15^ suggests that ICG localizes precisely to the tumor, we do not know the precise number of ICG-positive cells that can be detected by the average surgeon’s eye. This is also a potential area for future study.

Our assessment of margin adequacy was based on LR at 4 weeks after surgery. In the human condition, patients are typically evaluated for LR for many years postoperatively. It is entirely possible that, at a longer time point, the LR rate in group 1 animals (ICG guidance) would not be as favorable as we have presented here.

## Conclusion

We have described the use of ICG as an intraoperative guide for the removal of sarcoma in a validated orthotopic mouse model of OS. We appreciated that residual ICG signal, or the absence thereof, reliably predicted LR. Intraoperative ICG guidance led to decreased LR compared with animals whose surgeries were not guided by intraoperative ICG. Given these observations, the benign safety profile of ICG and its prior clinical use, we believe that the results of this study should be translated rapidly into the clinical realm.
